# Identification and Network-Enabled Characterization of Auxin Response Factor Genes in *Medicago truncatula*

**DOI:** 10.3389/fpls.2016.01857

**Published:** 2016-12-09

**Authors:** David J. Burks, Rajeev K. Azad

**Affiliations:** ^1^Department of Biological Sciences, University of North TexasDenton, TX, USA; ^2^Department of Mathematics, University of North TexasDenton, TX, USA

**Keywords:** auxin response factor, *Medicago truncatula*, co-expression networks, indole-3-acetic acid, legumes, nodulation

## Abstract

The Auxin Response Factor (ARF) family of transcription factors is an important regulator of environmental response and symbiotic nodulation in the legume *Medicago truncatula*. While previous studies have identified members of this family, a recent spurt in gene expression data coupled with genome update and reannotation calls for a reassessment of the prevalence of ARF genes and their interaction networks in *M. truncatula*. We performed a comprehensive analysis of the *M. truncatula* genome and transcriptome that entailed search for novel ARF genes and the co-expression networks. Our investigation revealed 8 novel *M. truncatula* ARF (MtARF) genes, of the total 22 identified, and uncovered novel gene co-expression networks as well. Furthermore, the topological clustering and single enrichment analysis of several network models revealed the roles of individual members of the MtARF family in nitrogen regulation, nodule initiation, and post-embryonic development through a specialized protein packaging and secretory pathway. In summary, this study not just shines new light on an important gene family, but also provides a guideline for identification of new members of gene families and their functional characterization through network analyses.

## Introduction

Phytohormone auxin, characterized by indole-3-acetic acid (IAA), is an important regulator of plant growth and development, including lateral root initiation, vascular differentiation, tropic responses, apical dominance, shoot elongation, and embryo patterning (Davies, 1995; Abel and Theologis, 1996; Guilfoyle et al., 1998). Recent studies have also shown that auxin plays an additional role in the organogenesis of nodules in leguminous plants (e.g., Suzaki et al., 2013), building on observations dating back to the 1930s when elevated levels of auxin were discovered in pea nodules (van Noorden et al., 2006). Despite significant recent advances in deconstructing the auxin signaling, only little is known of the auxin's role in nodulation. Nodule forming rhizobia can secrete bioactive auxin in addition to cytokinin and other signaling molecules such as nod factor, and it is believed that the combination of phytohormones and molecular signals work together to induce a local auxin accumulation in the rapidly dividing cortical cells of nodule primordia in several leguminous species (Boivin et al., 2016).

Genes referred to as early or primary auxin response genes can undergo transcriptional alterations within minutes of exposure to auxin, and fall into three major classes—*Aux/IAA*s, *SAURs*, and *GH3*s (Hagen and Guilfoyle, 2002). In many cases, the promoter regions of early auxin response genes contain one or more auxin response elements (AuxREs), distinguished by the conserved motif TGTCTC. A synthetic, palindromic TGTCTC AuxRE was used as bait in the yeast one-hybrid system that resulted in the discovery of the first auxin responsive transcriptional factor “auxin-response factor 1” (ARF1; Ulmasov, 1997). Since then, additional ARFs have been isolated with the capacity to either activate or repress transcription of down-stream target genes containing AuxREs in their promoter regions (Ulmasov et al., 1999a).

ARF proteins are typified by three conserved domains: an N-terminal B3-like DNA-binding domain (DBD), a C-terminal dimerization domain (CTD), and a middle region believed to be the activation or repression determinant domain depending on its amino acid sequence (Ulmasov et al., 1999b; Tiwari et al., 2003). Among these, the CTD domain appears to be the most optional and is sequentially similar to motifs III and IV of the Aux/IAA protein family (Ulmasov et al., 1999b); this domain is not present in certain ARF proteins including Arabidopsis AtARF3 and AtARF17 (Ulmasov et al., 1999a; Liscum and Reed, 2002). As a protein-protein interaction domain, the CTD domain allows the homo- and hetero-dimerization of ARFs and the hetero-dimerization of ARFs and Aux/IAA proteins (Kim et al., 1997; Ulmasov et al., 1999b; Wang et al., 2007). In the case of palindromic AuxREs, certain ARFs are stabilized in their binding to these motifs following dimerization with other ARFs (Ulmasov et al., 1999a).

For almost two decades, researchers performing functional analyses of the ARF gene family have attempted to uncover the full extent and significance of this important regulator of auxin-mediated growth and development. Much of our current understanding of the ARF family stems from research conducted in model plant *Arabidopsis*, within which the creation of *arf2* mutant alleles revealed AtARF2 as a pleiotropic developmental regulator (Okushima et al., 2005). Derepression of AtARF3, through the disruption of TAS3 trans-acting siRNAs, resulted in accelerated phase change and severe morphological and patterning defects of both leaves and floral organs (Fahlgren et al., 2006). It has been suggested that the functional redundancy of the ARF family is a reflection of its relative importance; only 4 of 18 single T-DNA insertion ARF mutants were shown to produce an obvious growth phenotype (Okushima et al., 2005); a double-null *Atarf6/Atarf8* mutation completely arrested flower development and repressed the production of jasmonic acid, whereas singular mutations in either *Atarf6* or *Atarf8* only slightly decreased self-fertilization (Nagpal, 2005); in regards to auxin transport, *Atarf7* mutants have no detectable phenotype, whereas *Atarf7* double and triple mutants associated with *Atarf16, Atarf17*, and *Atarf19* showed a significant reduction in auxin-dependent relocation of PIN-FORMED proteins, a family of transmembrane proteins that transport auxin as their substrate (Sauer et al., 2006).

Leguminous plants, such as, *Medicago truncatula*, are known to form symbiotic relationships with soil bacteria, namely rhizobia, by developing specialized root nodules to house the bacteria that fix molecular nitrogen into ammonia in exchange for the carbohydrate byproducts of photosynthesis (Long, 1989). Central to this symbiosis is auxin, whose polar transport and interplay with cytokinin are believed to be indispensable in proper nodule formation and development (Liu et al., 2015; Shen et al., 2015). Prior to nodule initiation in indeterminate legumes such as *M. truncatula*, acropetal auxin transport is inhibited at the initiation site, and auxin-responsive reporter gene expression localizes in the dividing inner and outer cortical cells (Mathesius et al., 1998; van Noorden et al., 2007). The concentration of auxin has an inverse relationship with the number of nodules produced in *M. truncatula* roots (van Noorden et al., 2006), and the presence of auxin transport inhibitors induces the formation of pseudonodules (Rightmyer and Long, 2011).

As an integral component of auxin-regulation, the ARF gene family encodes proteins that regulate nodule formation and development in *M. truncatula* (Breakspear et al., 2014; Shen et al., 2015). More specifically, the role of ARFs in the regulation of symbiosis has been confirmed through functional characterization, with orthologous mutant *arf16a* resisting *Sinorhizobium meliloti* (*S. meliloti*) infection (Breakspear et al., 2014), and thus further exemplifying auxin's role in the initiation of nodules. Identification and analyses of *M. truncatula* ARFs (MtARFs) are thus critical for understanding the biological processes governed by ARF transcription regulation, and for elucidating, in particular, the relationship between auxin and nodulation. Computational approaches have been utilized to identify putative ARF genes in rice (*Oryza sativa*), field mustard (*Brassica rapa*), poplar (*Populus trichocarpa*), and tomato (*Solanum lycopersicon*) based on *A. thaliana* ARF (AtARF) gene family members discovered through both functional and *in silico* genomic analysis (Hagen and Guilfoyle, 2002; Kalluri et al., 2007; Wang et al., 2007; Zhang et al., 2012; Zouine et al., 2014). Specifically, in *M. truncatula*, genome analysis has helped identify several putative members of MtARF gene family, and their associated expression patterns following infection by *S. meliloti* (Shen et al., 2015). Yet a comprehensive study of the currently available genome and transcriptome data is clearly needed to catalog putative members of the MtARF gene family and understand their significance through a systems-level analysis. Our attempt to perform such an analysis led to the discovery of several novel, yet uncharacterized MtARF genes, which were further probed for their potential functions using weighted and unweighted co-expression network analysis.

Given the regulatory nature of the MtARF family, deciphering their interactivity at a systems-level can be highly informative. Therefore, after identifying novel MtARF genes, we constructed a genome-wide gene co-expression network using expression data from over 700 high quality microarrays. While this type of network has been constructed specifically for other model plants, such as, *Arabidopsis* (Mao et al., 2009), a *Medicago* gene co-expression network (MGCN) has never been formally studied. Similar to that in *Arabidopsis*, the gene co-expression networks in *Medicago* were also found to be modular in this study, thus enabling assessment of functional roles of MtARFs via reconstruction of gene functional modules using statistical methods. We employed an empirical, top-down clustering approach to module identification and the clusters with MtARFs were further examined for any functional or metabolic conservation. By exploiting the topological properties of three independent co-expression networks, we have created a novel and potentially more informative protocol for the functional characterization of the newly discovered members of a gene family. In addition to an all-encompassing MGCN, we also constructed a root-specific MGCN to understand the role of MtARFs in the establishment of nodulation and symbiosis using a weighted scale-free network built on the Weighted Gene Coexpression Network Analysis (WGCNA) R package (Langfelder and Horvath, 2008), and a Mutual Rank network using weighted Pearson correlation coefficient (PCC) calculations. In what follows we describe our approach and results and conclude with a discussion on the significance and further implications of this study.

## Materials and methods

### Identification of *M. truncatula* ARF genes

An updated version (v1) of the Mt4.0 genome release of *M. truncatula* was used to identify MtARF gene family members, utilizing approximately 360 Mb of sequences of which only 70% were represented in the prior Mt3.5 release (Tang et al., 2014). A multi-tiered search was performed for a robust and sensitive detection of MtARF genes. First, all 23 *Arabidopsis* ARF protein sequences were downloaded from The Arabidopsis Information Resource (TAIR), and used as BLAST queries for NCBI BLAST 2.2.28 against the Mt4.0v1 genome (Altschul et al., 1990; Lamesch et al., 2012). An *e*-value cutoff of 10^−3^ was used due to the relatively high similarity of the query and subject organisms. Results were filtered to remove redundant hits, and subjected to domain analysis using the Pfam profiles associated with ARF gene family: Pfam 02309: AUX_IAA; Pfam 02362: B3; Pfam 06507: Auxin_resp. Pfam domain 02309 is considered an optional domain; Predicted MtARFs lacking either the Pfam domains 02362 and 06507 were removed from the prediction list. An additional query of the Mt4.0v1 proteome was conducted using the three aforementioned Pfam domains. Both phytozome (http://www.phytozome.net/) and JCVI (http://www.jcvi.org/cms/home/) were used to conduct the domain search, and Pfam domain 02309 was again considered an optional component (Goodstein et al., 2012). Results from the above two searches were combined and duplicate hits were filtered from the final dataset.

### RNA-seq expression analysis of MtARF genes

Expression analysis was conducted using publically available RNA-Seq data from the NCBI Sequence Read Archive (SRA). Raw RNA-Seq data for 36 samples of wild-type A17 total RNA extractions, were converted to FASTQ format using fastq-dump of the SRA Toolkit (http://hannonlab.cshl.edu/fastx_toolkit/). Quality assessment and adapter identification of each read was performed using FastQC (http://www.bioinformatics.babraham.ac.uk/projects/fastqc/). Subsequent adapter removal and trimming was conducted using Trimmomatic (http://www.usadellab.org/cms/?page=trimmomatic). Processed reads were then aligned to the Mt4.0v1 *M. truncatula* genome using Tophat 2.1.0 (Trapnell et al., 2009). Alignment files generated by Tophat were then processed using Cufflinks 2.2.1 to obtain normalized count of paired-end reads mapping onto each gene, defined as fragments per kilobase of transcript per million mapped reads (FPKM).

Additional expression analysis was conducted using the JCVI online genome browser, JBrowse (Skinner et al., 2009). This regularly updated online RNA-Seq coverage browser includes expression profiles for Mt4.0v1 gene models based on publically available RNA-Seq data generated from blade, bud, nodule, open flower, seedpod, and root tissues. Only MtARF gene models with significant expression in at least a single tissue type were considered for further analysis.

### Unweighted network generation and module identification

The February 2015 V3 release of the Medicago Gene Expression Atlas (MtGEA), comprising 739 arrays over 274 experiments, was used in its entirety to generate a *M. truncatula* co-expression network (Benedito et al., 2008; He et al., 2009). Gene expression data in the MtGEA has been normalized, and was not adjusted for further analysis. Probe-set IDs were converted to their associated International Medicago Genome Annotation Group (IMGAG) IDs.

Genes were selected for the network based on two requirements: (1) the ratio between the standard deviation and mean of the gene's expression values over the entire 739 array dataset is greater than or equal to 0.5; (2) the difference between a gene's maximum and minimum expression values over the entire dataset is >32. These criteria were selected based on a prior work on *A. thaliana*, and were adapted here for *M. truncatula* in order to remove genes with little variation in expression that would unnecessarily inflate the number of highly correlated genes (Mao et al., 2009). Genes that passed these filters were then log_10_ transformed, with any negative transformations being set to 0. PCC for every combination of two genes was calculated using the log_10_ transformed values (Liu, 2015). Using custom scripts written in Python 3.4, network comparison across various PCC cutoffs was performed to find the PCC cutoff that minimizes the network density, as is standard practice for maximizing clustering potential (Mochida et al., 2011; Feng et al., 2012; Liang et al., 2014). Network density is defined as the ratio of the number of edges with PCC values above the cutoff to the number of all possible edges between nodes in a given network (Mao et al., 2009).

Edges and nodes of the co-expression network were analyzed using the Markov Clustering (MCL) algorithm to identify the functional modules (van Dongen, 2000), as was done similarly for *A. thaliana* in a previous study (Mao et al., 2009). Inflation values ranging from 1 to 5 were tested at increments of 0.1, and the resultant clusters were analyzed for GO term enrichment in order to determine the parameter setting at which the GO term enrichment for the functional modules is maximal (Mao et al., 2009; Liu et al., 2014). Inflation parameter values were assessed for the maximum number of enriched clusters that could be generated for the network (i.e., clusters with significant term enrichment in any of the three GO sub-ontologies, with enrichment assessed against the whole network background). An optimal setting was thus found at the inflation parameter value of 1.2.

The process was repeated, now only using the root data from the MtGEA. As with the full network, the root data were subjected to the same filters, and the optimal network was obtained based on the PCC cutoff that minimized the network density.

For the unweighted networks, the clustering coefficient for a node *n* was obtained as Mao et al. (2009):

$$\begin{array}{l} C_{n}=2e_{n}/\left(k_{n}\left(k_{n}-1\right)\right) \end{array}$$

where *k*_*n*_ is the number of direct neighbors of *n* and *e*_*n*_ is the number of connected pairs among all neighbors of *n*. For networks, the clustering coefficient is obtained as the average over all node clustering coefficients in the network. Here, a node refers to a gene, and its degree refers to the number of connections that the gene has with other genes of the network based on the PCC cutoff. Genes with a PCC above the threshold are connected by an unweighted edge, while PCC values below the threshold are not considered for further analysis.

### Weighted network generation and module identification

Due to the relatively strict PCC cutoffs imposed by the unweighted network analysis, a weighted network model was also built using the Weighted Correlation Network Analysis (WGCNA) package in R (Langfelder and Horvath, 2008). The entire February 2015 V3 release of the MtGEA was used. Prior to analysis, the expression values for probe-set IDs included in MtGEA were checked for missing values and the experiments were clustered in order to identify any outliers within the dataset.

Next, pairwise Pearson correlations were obtained for genes in the atlas, and subsequently raised to the lowest power β for which an adjacency matrix consisting of these raised correlation values satisfies a scale-free network topology (Langfelder and Horvath, 2008). That is, the connection strength *a*_*ij*_ for nodes *x*_*i*_ and *x*_*j*_ was obtained as *a*_*ij*_ = |corr(*x*_*i*_, *x*_*j*_)|^β^. In order to satisfy a scale-free topology, WGCNA requires that the frequency distribution of the connectivity approximate a power-law distribution (i.e., a linear relationship on log scale; Langfelder and Horvath, 2008). Co-expression modules were determined using the “dynamic tree cut” hierarchical clustering algorithm included with WGCNA.

### Mutual rank network generation and direct neighbor analysis

Due to high values of network PCC cutoffs coupled with comparatively low correlation values across the MtARF family, a rank-based method (Obayashi and Kinoshita, 2009) was also used in an effort to produce a network with more MtARF members present. First, the entire MtGEA was weighted based on sample redundancy. Whereas, the MtGEA offers a wide variety of microarray data sources, finding a suitable PCC cutoff based on network density results in a large amount of information loss. To ensure that the abundance of highly correlated gene pairs responsible for the stringent cutoffs are not due to sample redundancy within the MtGEA, a weighted PCC approach was used according to Obayashi and Kinoshita (2009). First, the PCC between samples in each possible pair, say *Sa* and *Sx*, is calculated and referred to as the sample-to-sample similarity *J*_*Sa, Sx*_. Similarity values below 0.4 were set to 0 to ensure that significantly dissimilar samples were given an increased weight, as opposed to more similar samples with similarities above 0.4. For similarities above 0.4, the adjusted sample-to-sample similarity $J_{sa,sx}^{\prime }$ = (*J*_*Sa, Sx*_ − C)/(1−C), where C is the 0.4 cutoff threshold mentioned above. The total sample redundancy for a sample *Sa* is the summation of sample *Sa*'s sample-to-sample similarities with all other samples in the dataset, $J_{sa}^{\prime }=\sum_{Sx}J_{sa,sx}^{\prime }$. Because high redundancy samples should be weighted less in the gene-to-gene PCC calculations, the weight of a sample *S*_*a*_, *W*_*Sa*_, is defined as the inverse of the square root of sample *Sa*'s redundancy. The weighted PCC for any two genes, *g*1 and *g*2, thus becomes (Obayashi and Kinoshita, 2009; Obayashi et al., 2011):

$$\begin{array}{l} \frac{\sum_{s}W_{s}\left(RE_{g1,s}-\bar{RE_{g1}}\right)\left(RE_{g2,s}-\bar{RE_{g2}}\right)}{\sqrt{\sum_{s}W_{s}{\left(RE_{g1,s}-\bar{RE_{g1}}\right)}^{2}\sum_{s}W_{s}{\left(RE_{g2,s}-\bar{RE_{g2}}\right)}^{2}}}, \end{array}$$

where $\bar{RE_{g1}}$ and $\bar{RE_{g2}}$ are the weighted average relative expression values of genes *g*1 and *g*2, respectively, defined as:

$$\begin{array}{l} \bar{RE_{gx}}=\;\frac{\sum_{s}W_{s}RE_{gx,s}}{\sum_{s}W_{s}}. \end{array}$$

The Mutual Rank (MR) for any two genes is derived from their weighted PCC values (Obayashi and Kinoshita, 2009; Obayashi et al., 2011). First, every weighted PCC for a given gene is ranked in the order of correlation (high to low). This rank, ranging from 1 to *n*−1 for *n* genes in the dataset, is then compared to the ranked list of weighted PCC values for a second gene. Their mutual rank is derived as the geometric average of their corresponding ranks. For example, two genes known as gene A and gene B, would have a MR derived as follows (Obayashi and Kinoshita, 2009; Obayashi et al., 2011):

$$\begin{array}{l} MR\left(AB\right)=\sqrt{Rank_{A\to B}\;*\;Rank_{B\to A}} \end{array}$$

**Figure 1:**
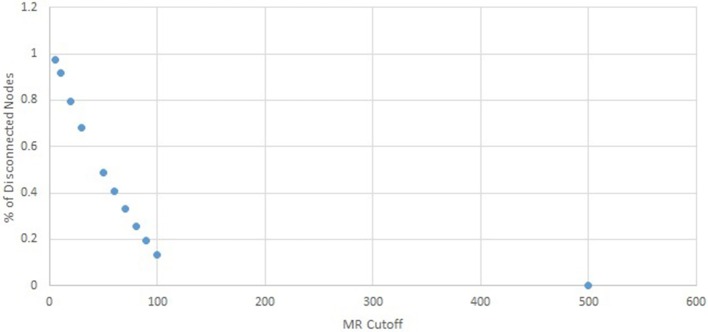
**Percent disconnected nodes in the MR network as a function of MR cutoff**. A cutoff of 100 was chosen, as further increase in cutoff results in insignificant decrease in disconnected nodes.

To find a suitable cutoff for the *MR* network, the percentage of disconnected nodes was identified at increasing *MR* cutoffs. A cutoff of 5 resulted in over 97% of the network's nodes being completely disconnected, which rapidly decreased to 68% at a cutoff of 30. The relationship between MR cutoff and percentage of disconnected nodes is shown in Figure 1. A cutoff of 100 was chosen, which resulted in only 13.2% of the network's nodes being disconnected. Using an MR below this value could result in unnecessary information loss, whereas higher *MR* cutoffs give only minimal increase in connected nodes.

### Sequence similarity and gene expression visualization

Sequence similarity between ARF protein sequences in *A. thaliana* and *M. truncatula* was visualized using Circos through the Circoletto web interface (Darzentas, 2010). The hit threshold was adjusted to 1E-100 due to high level of sequence conservation. *M. truncatula* genes were used as the query against a database of *A. thaliana* ARF genes. Only the best hit per query was selected, with all local alignments per best hit diagramed. Multiple-sequence alignment of the MtARF family was performed using Clustal Omega, and visualized with the ETE3 framework (Sievers et al., 2011; Huerta-Cepas et al., 2016).

All protein sequences for *Arabidopsis* were downloaded as part of the TAIR v10 release from The Arabidopsis Information Resource, and converted to a BLAST-compatible protein database using the makeblastdb function of NCBI BLAST+ 2.2.28 (Altschul et al., 1990; Lamesch et al., 2012). This process was repeated using the Mt4.0v1 protein sequences downloaded from JCVI (Tang et al., 2014). BLASTp analysis was performed using the MtARF and AtARF protein sequences as queries against their respective protein database, and best-hits were collected based first on *E*-value, and in the event of ties, by bitscore. Best-hits between the two families were then compared, and reciprocal best-hits were recorded as potential orthologous genes.

A heatmap for visualizing expression of all available MtARFs within the MtGEA was created using the “aheatmap” function of the NMF library (Hoyer, 2004). Euclidean clustering was used, and the average expression across all probeset IDs for a given MtARF were used in cases where multiple probes mapped to a single gene model. Prior to visualization, expression values were log_10_ transformed.

### Motif analysis and domain identification of MtARF proteins

MtARF protein sequences were analyzed for novel domains using the Multiple EM for Motif Elicitation (MEME) web server (Bailey et al., 2009). Default parameters were used with the normal discovery mode. Each protein sequence was then analyzed for known domains using the Pfam database hosted by the European Bioinformatics Institute (Finn et al., 2015). A batch search was performed using an *E*-value cutoff of 1.0. Domains discovered through the Pfam search algorithm were compared to the motifs identified by MEME in each protein sequence to determine whether or not any novel domains were present in the gene family.

### Physiochemical analysis of MtARF proteins

Analysis to determine physiochemical properties of all MtARF protein sequences was conducted *en masse* using the ProtParam module of the SeqUtils package of Biopython 1.66 (Cock et al., 2009). Protein molecular weights, instability indices, isoelectric points, and amino acid composition were all determined using the ProteinAnalysis class.

### Tanglegram

Dendrograms from the MtARF heatmap and multiple-sequence alignment analysis were imported into Dendroscope 3.5.7, and the built-in tanglegram algorithm was used to generate cross-linkages between the two trees (Huson and Scornavacca, 2012).

## Results

### Identification of MtARFs

Using annotated AtARF protein and nucleotide sequences obtained from TAIR, 86 unique MtARF candidates were identified in the Mt4.0v1 genome release, as described in Section Materials and Methods. These 86 candidates were scanned for conserved domains, and their transcriptional activities were assessed using publicly available expression data to filter out gene models showing no expression. An additional search of the Mt4.0v1 proteome was conducted based on the conserved domains of the MtARF family to ensure that no genes sharing this combination of domains were missed. A total of 22 MtARF genes, with signature conserved domains and significant transcriptional activities, were thus identified and given temporary names based on their chromosomal locations. Of these MtARFs, 14 were previously identified in a similar study conducted in 2015 (Shen et al., 2015), and the remaining 8 are our novel discoveries. In Table 1 we provide information about each of the 22 MtARF genes, including chromosomal location, ORF length, intron count, polypeptide length, molecular weight, and isoelectric point.

**Table 1:** **IMGAG ID, chromosomal location, and physiochemical properties of MtARFs**.

**MtARF**	**IMGAG**	**Chromosomal location**	**CDS length**	**Peptide length**	**Introns**	**Molecular weight**	**Isoelectric point**	**Instability index**
MtARF1	Medtr1g024025	chr1:7778460-7771119	3798	1265	21	141162.1444	5.264343262	53.24634783
MtARF2	Medtr1g064430	chr1:28345468-28349210	2109	702	3	78527.0301	6.836853027	51.62905983
MtARF3	Medtr1g094960	chr1:42729587-42732600	1860	619	1	68962.9542	7.53314209	50.43781906
MtARF4	Medtr2g005240	chr2:92911-103331	2016	671	13	74979.3898	5.714660645	66.57497765
MtARF5	Medtr2g014770	chr2:4274889-4270421	2049	682	10	74200.528	6.112243652	51.10498534
MtARF6	Medtr2g018690	chr2:5912688-5906356	2727	908	13	100825.9555	6.139831543	63.10067181
MtARF7	Medtr2g043250	chr2:18843526-18835022	3345	1114	13	124368.6771	5.99005127	71.5888061
MtARF8	Medtr2g093740	chr2:39983867-39979955	2472	823	11	91728.6073	6.854064941	51.71447145
MtARF9	Medtr2g094570	chr2:40353672-40350058	2268	755	5	84007.2728	8.641296387	42.81234437
MtARF10	Medtr3g064050	chr3:28806780-28798989	2550	849	13	94265.7491	5.923400879	56.86090695
MtARF11	Medtr3g073420	chr3:33106580-33110521	1782	593	3	64872.5714	6.151672363	40.00421585
MtARF12	Medtr4g021580	chr4:5935075-5939270	2004	667	13	74193.6064	6.220397949	49.73314843
MtARF13	Medtr4g058930	chr4:21714903-21719127	2097	698	3	77244.7297	7.207824707	42.71091691
MtARF14	Medtr4g060460	chr4:22234433-22239989	2379	792	11	88097.6377	6.36138916	52.94357323
MtARF15	Medtr4g088210	chr4:34796152-34801111	2139	712	9	79087.7449	6.499206543	49.80716292
MtARF16	Medtr4g124900	chr4:43700709-43706464	3363	1120	12	125413.3046	6.077453613	66.12108036
MtARF17	Medtr5g076270	chr5:32499847-32489763	2526	841	13	93281.6568	5.929992676	57.34887039
MtARF18	Medtr7g062540	chr7:22756278-22761697	2043	680	12	76164.5327	5.74407959	55.89339706
MtARF19	Medtr8g027440	chr8:9733998-9728759	2034	677	13	75734.7637	6.175354004	52.74669129
MtARF20	Medtr8g079492	chr8:34084030-34077887	2748	915	13	102333.3159	6.080993652	63.69246995
MtARF21	Medtr8g100050	chr8:40230869-40235830	2502	833	12	93074.1597	6.064147949	56.99292917
MtARF22	Medtr8g101360	chr8:42569517-42563540	3291	1096	13	121073.0802	6.226623535	62.48551095

### Chromosomal distribution of MtARF genes

**Figure 2:**
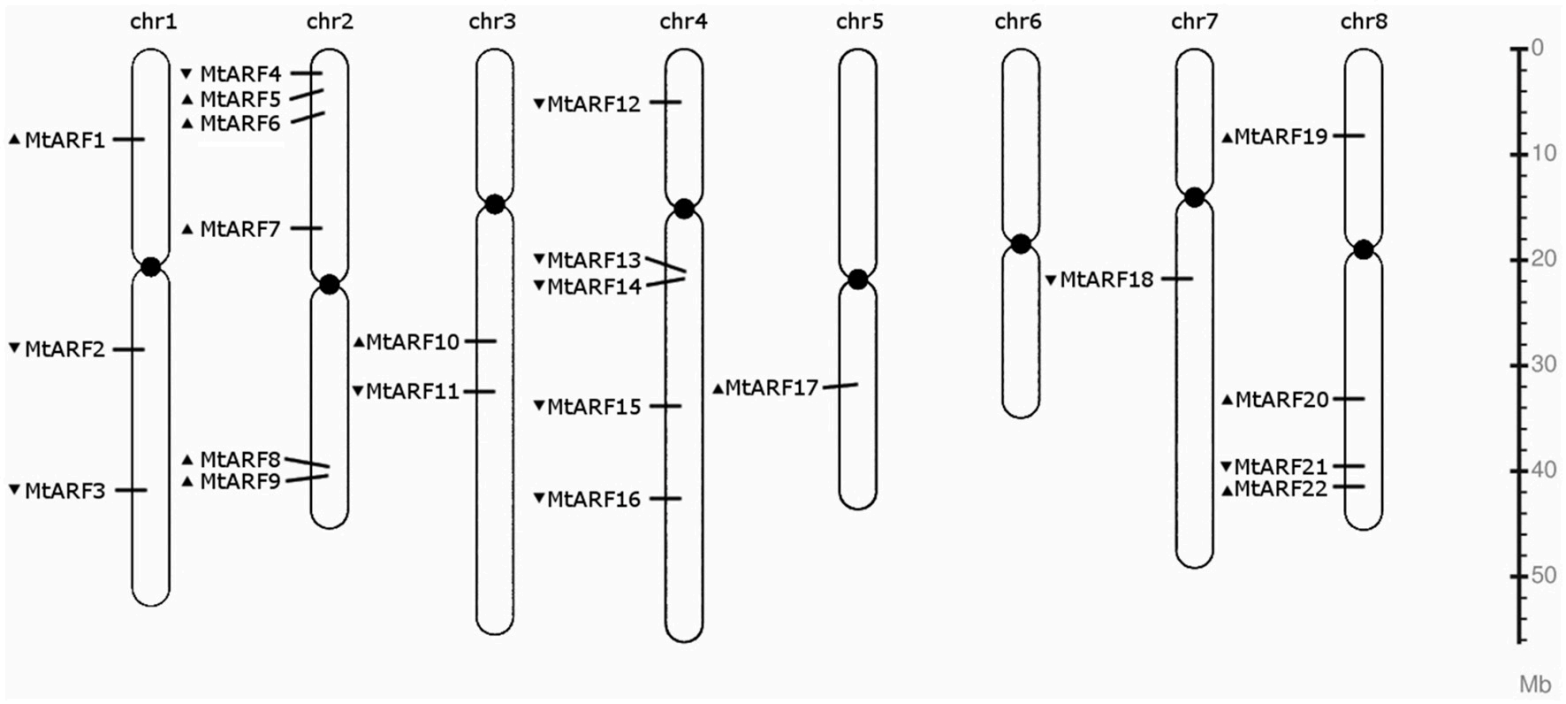
**Chromosomal location map of the 22 MtARF genes**. Arrow before a gene name indicates the coding polarity of the gene.

All but one of *M. truncatula*'s eight chromosomes host at least one MtARF gene (Figure 2). Chromosome 6 does not harbor an MtARF gene. MtARF family is most represented on chromosomes 2 and 4, with six (27%) genes on chromosome 2 and five (23%) genes located on chromosome 4, followed by chromosome 8 containing four MtARF genes, chromosome 1 containing three MtARFs, chromosome 3 harboring two MtARF genes, and chromosomes 5 and 7 containing one MtARF gene each.

### Motif analysis and protein parameter analysis of MtARFs

**Figure 3:**
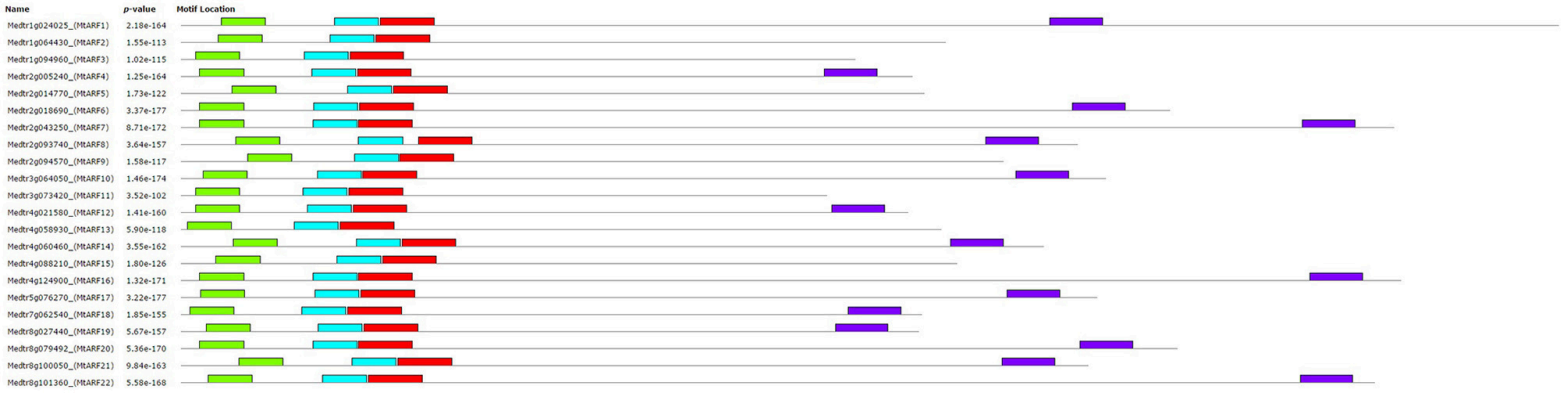
**Motifs identified in predicted MtARFs by MEME**. Motifs are shown as colored boxes. Aux_resp domain is represented by two motifs (“teal” and “red”). The carboxy-terminal motif (“purple”), representative of the optional AUX_IAA domain, is only present in 15 MtARF sequences.

The MEME suite was used to identify motifs in each of the 22 MtARF proteins. Altogether, four different motifs were isolated from MtARF family. Using the Pfam database, Motif 3 and Motif 4 were identified as the B3 DNA binding (PF02362.18) and AUX_IAA (PF02309.13) domains, respectively. Motif 1 and Motif 2 were each representative of a portion of the Auxin_resp (PF06507.10) domain. The domain locations in each MtARF, as identified from their respective motifs, are shown in Figure 3. Of the 22 MtARFs, 15 contained the carboxy-terminal AUX_IAA domain, with 7 of these 15 also containing a glutamine-enriched middle-region thus inferring their roles as transcriptional activators. All 22 MtARF proteins contained motifs representing the DNA-binding B3 and “Auxin_resp” domains. The 7 MtARF proteins (MtARF: 2, 3, 5, 9, 11, 13, 15) that did not contain a carboxy-terminal AUX_IAA domain did contain relatively higher percentages of serine and glycine residues in their middle region, suggesting their potential role as transcriptional repressors. The percentages of Q, S, and G amino acids for each MtARF are given in Table 2.

**Table 2:** **Percent glutamine (Q), serine (S), or glycine (G) in each MtARF protein sequence**.

**MtARF**	**%Q**	**%S**	**%G**
MtARF1	5.924171	12.5	5.946602
MtARF2	4.409673	12.15227	8.931186
MtARF3	4.354839	9.583333	5
MtARF4	3.869048	11.92146	7.713885
MtARF5	3.660322	10.93892	5.834093
MtARF6	8.580858	8.726753	8.583691
MtARF7	14.08072	11.9912	5.940594
MtARF8	4.368932	10.94276	7.744108
MtARF9	3.703704	11.90476	6.25
MtARF10	9.176471	11.52019	7.244656
MtARF11	3.535354	10.31746	7.275132
MtARF12	5.08982	10.49327	5.650224
MtARF13	3.433476	8.819346	6.970128
MtARF14	4.035309	11.53239	5.21327
MtARF15	4.347826	11.2232	7.061791
MtARF16	14.27297	11.97605	4.94012
MtARF17	8.07601	10.88314	5.084746
MtARF18	5.286344	11.12903	6.451613
MtARF19	4.719764	9.439528	5.60472
MtARF20	10.80786	10	7.764706
MtARF21	5.035971	10.86637	5.139501
MtARF22	9.662716	11.15108	6.115108

Physiochemical analysis of the MtARF protein sequences unveiled a highly variable molecular weight ranging from ~26,000–141,000 Da. Isoelectric point was more conserved, averaging 6.35 with a standard deviation of 0.72 across the 22 proteins. Additional physiochemical properties for the MtARF proteins are given in Table 1.

### Relationships between AtARFs and MtARFs

**Figure 4:**
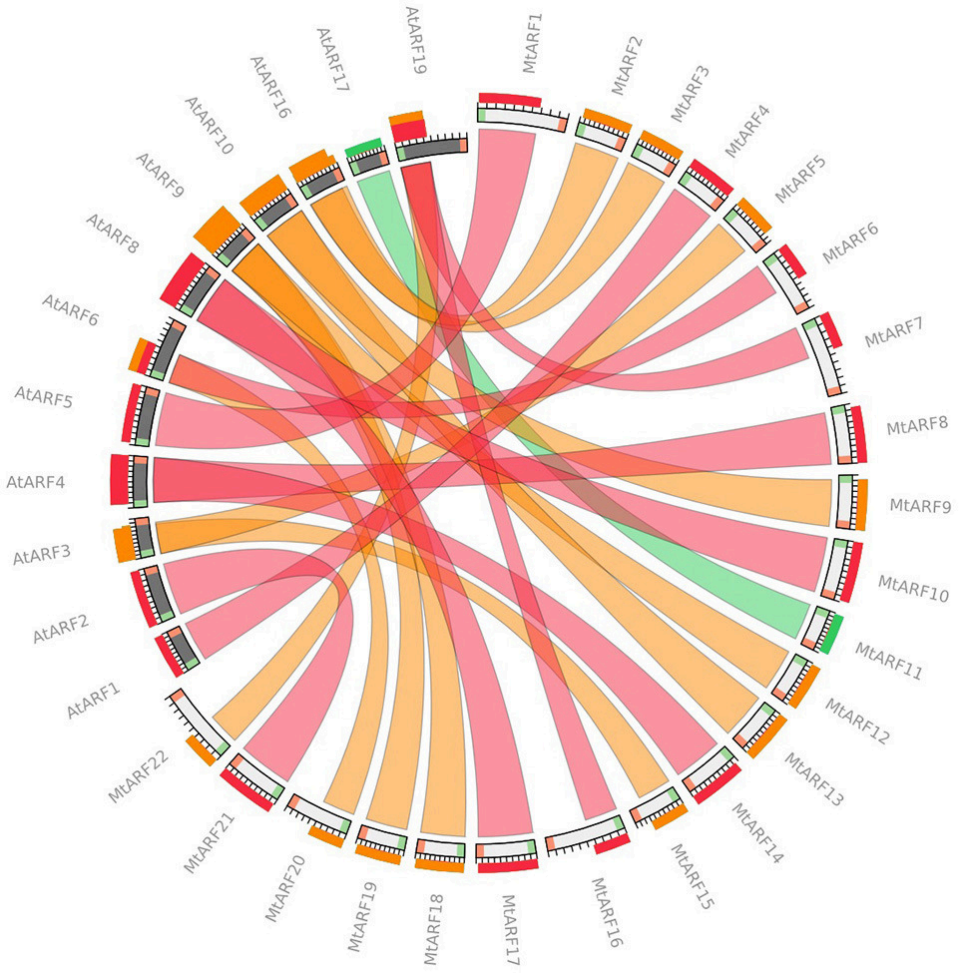
**Circoletto radial diagram linking the *M. truncatula* and *A. thaliana* ARF orthologs with ribbons**. Colors of the ribbons are relative to the best BLAST alignment score, with matches within 25% of the best match as red, within 50% as orange, and within 75% as green. White (MtARF) and black (AtARF) bands on the periphery of the diagram represent the protein sequence, with the start and end of the sequence shown as green and red blocks, respectively. The terminal ribbon width corresponds to the portion of sequence aligned by BLAST (shown as sequence band).

Circoletto, a web interface for comparing two sequence libraries via Circos, was used to identify and visualize the *A. thaliana* ARF protein sequences and their closest MtARF ortholog. An *E*-value of 1e-100 was used, and only the best match between the subject (MtARF) and query (AtARF) sequences was considered (Figure 4). AtARF4 and AtARF19 produced the highest number of best-matches with 3 of the MtARF sequences each (Figure 4).

Reciprocal best-hit analysis was also performed to identify orthologous ARFs between both the species. Protein databases generated for both plants' proteomes were used for performing reciprocal BLAST analysis. Starting with *A. thaliana* ARFs as queries and performing two-way BLAST, a total of 11 reciprocal best blast hits were identified between *M. truncatula* and *A. thaliana* (Table 3). Of these, 5 were found on chromosome 2 with the remaining 6 are distributed on chromosomes 1, 3, 4, 5, and 8. These results correspond to the bands generated by Circoletto (Figure 4). It should also be noted that all 23 AtARF best-hits were to one of the 22 MtARFs identified in this study, with 10 AtARF best-hits pairing with Medtr4g021580 (MtARF12).

**Table 3:** **Reciprocated best-hits between the *M. truncatula* and *A. thaliana* ARF families using blastp analysis and their respective protein databases**.

**MtARF**	**AtARF**
MtARF5	AtARF3
MtARF12	AtARF9
MtARF16	AtARF19
MtARF4	AtARF1
MtARF1	AtARF5
MtARF8	AtARF4
MtARF9	AtARF10
MtARF21	AtARF2
MtARF6	AtARF6
MtARF17	AtARF8
MtARF11	AtARF17

### Expression heatmap

**Figure 5:**
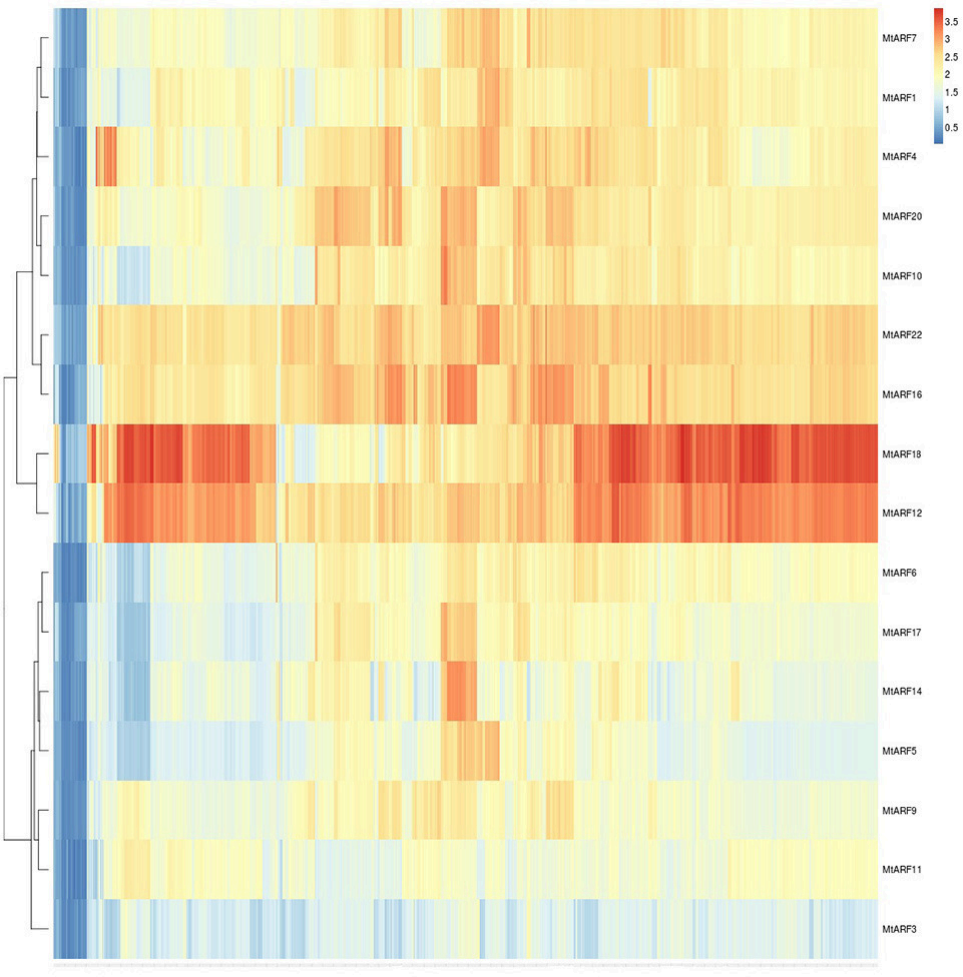
**Gene expression heatmap for all 16 MtARF genes that are represented by at least one probe in the MtGEA**. Hierarchical clustering was performed using the *aheatmap* module of the NMF R library.

A heatmap of MtARF genes depicting their expression change across the entire gene atlas was created based on Euclidean distance (Figure 5). Sixteen MtARF gene models were represented by one or more probes within the MtGEA, with several having multiple probe IDs. Upon hierarchical clustering, the16 MtARFs formed major clades. Interestingly, all 4 MtARFs lacking the optional Aux/IAA domain formed a distinct group, with noticeably lower overall expression across the entire dataset, possibly establishing a link between average expression and the role of a given MtARF. The 5 MtARFs with the highest percentage of glutamine-residues (Table 1) were found in the adjacent clade, further lending credence to the notion of transcriptional activation by MtARFs with higher overall expression compared to MtARF repressors.

### Unweighted gene co-expression network topology

**Figure 6:**
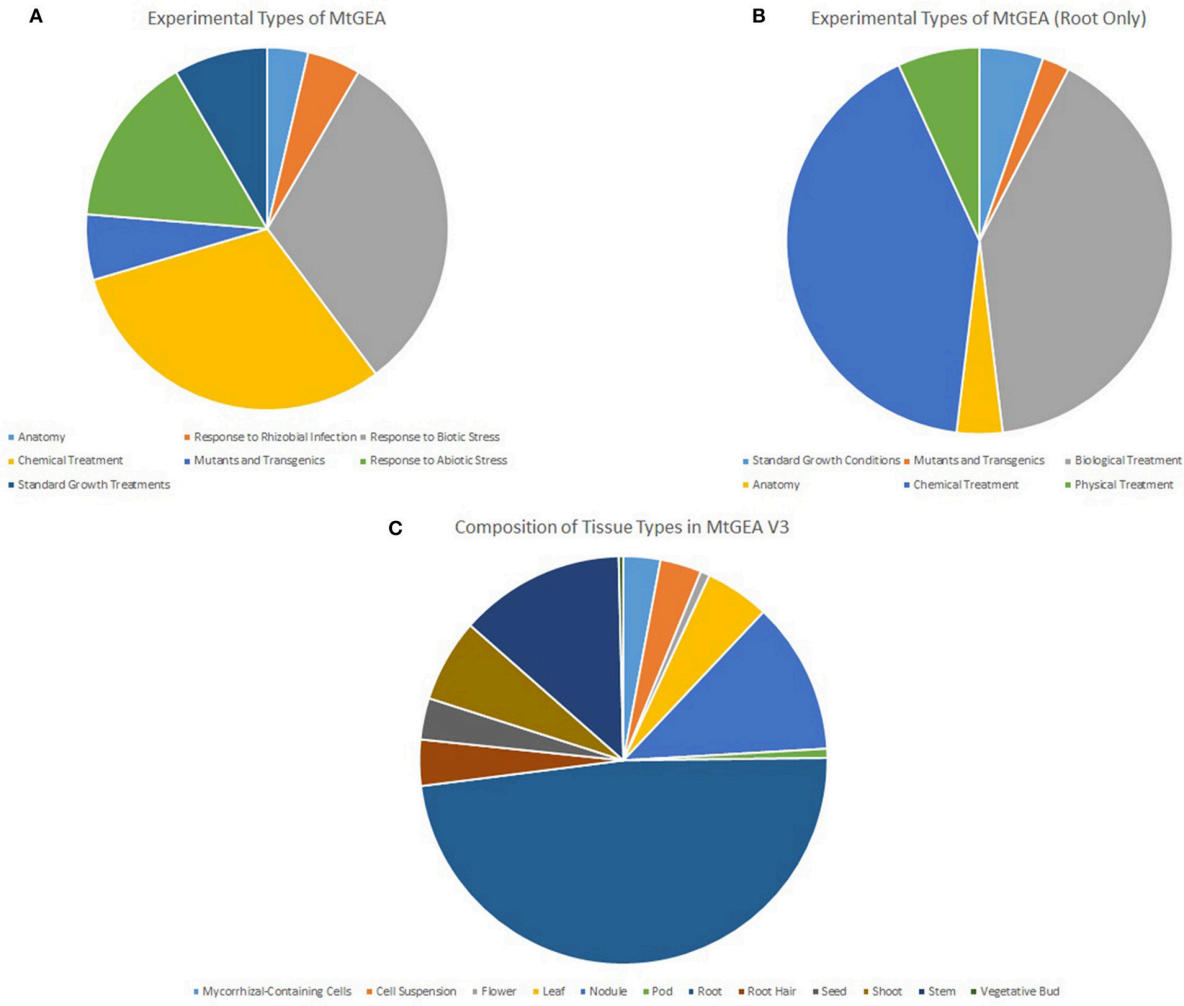
**Tissue and experimental types represented in MtGEA V3**. Data representing the whole atlas **(A)** and the root only atlas data **(B)** were used to generate the full and root MGCNs, respectively. Root tissue comprised nearly half of the entire MtGEA **(C)**.

To generate the full MGCN, all 739 arrays originating from 274 experimental conditions were used to calculate pair-wise correlations between genes. Arrays included in the V3 release of the MtGEA have been normalized, and were used as-is for further analysis. Figure 6A shows the experimental conditions represented amongst the 739 arrays; the experimental conditions present in the root-only dataset are shown in Figures 6B,C shows the specific tissue types studied. The degree of diversity present in the gene atlas allows for a reliable inference of co-expression relationships amongst the gene pairs.

To reduce noise and avoid unnecessary inflation, only genes with significant changes across the 739 arrays were used (see Section Materials and Methods). Gene expression values were log-transformed, and the PCCs was calculated for all possible gene pairs. Probeset IDs were mapped to their corresponding IMGAG IDs, and any pairs with identical gene models were discarded from further analysis. Altogether, 16,991 individual Mt4.0v1 gene models were found to be represented in the atlas.

**Figure 7:**
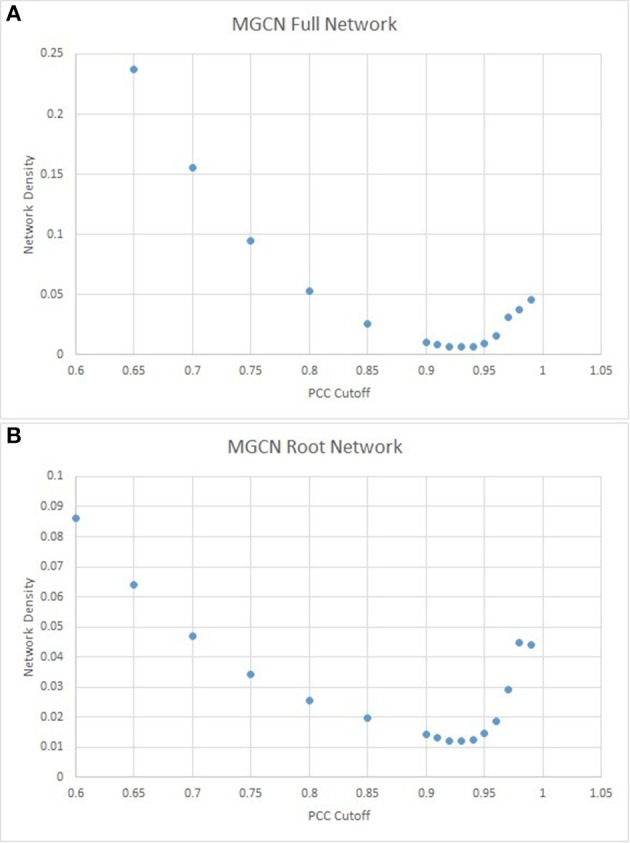
**Network density as a function of PCC cutoff for the full (A)** and root **(B)** MGCN.

Network density was calculated for PCC cutoffs ranging from 0 to 1 (see Section Materials and Methods). As shown in Figure 7A, the network density achieves a minimum at a PCC cutoff of ~0.93. This cutoff is much more stringent in comparison to similarly constructed networks in *Arabidopsis* and *Vitus* (Mao et al., 2009; Liang et al., 2014), and therefore much of the potential information present within the network is prone to be lost in the efforts to minimize the noise. In this network configuration of 68,428 surviving edges, only 4257 (8.35%) of all Mt4.0v1 gene models are present at a network density of 0.0075.

Given the functional significance of MtARF gene family in nodulation, a secondary network using only the 132 root arrays from the MtGEA was also constructed. As before, the PCC cutoff was established via network density minimization (Figure 7B). Similar to the full atlas network, the root-only network had an identical PCC cutoff at 0.93, with 42,855 surviving edges, 2663 surviving nodes, and a network density of 0.0121.

**Figure 8:**
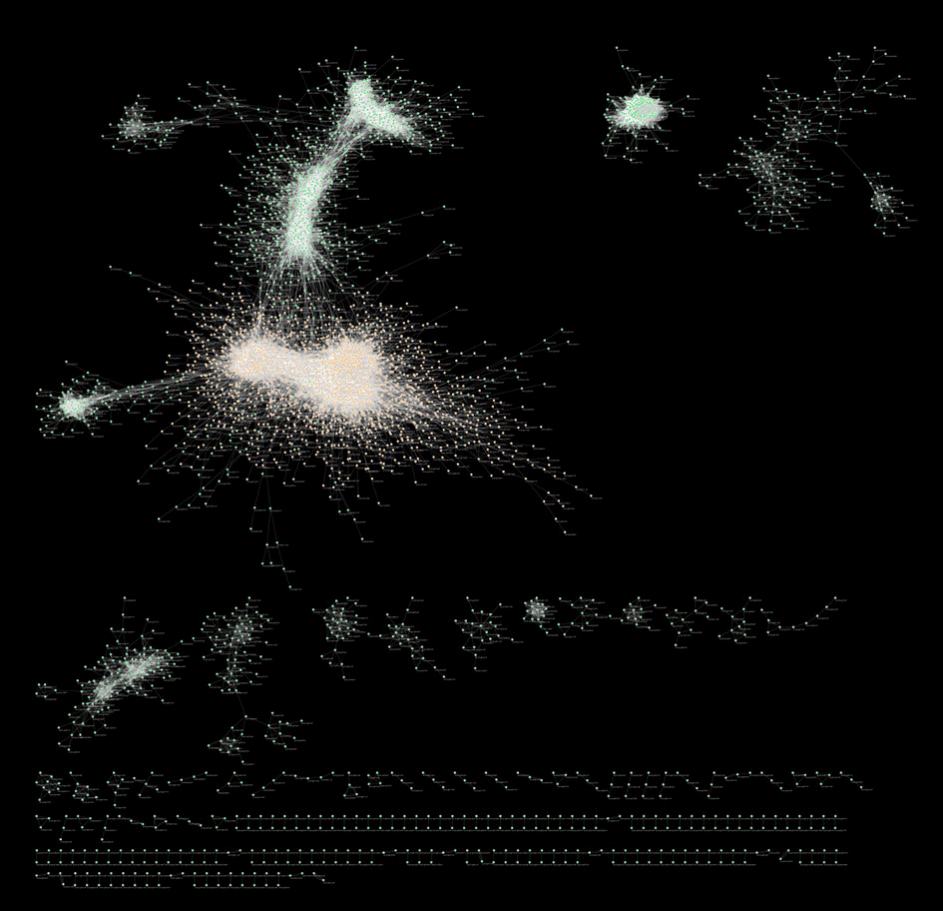
**Cytoscape visualization of the full MGCN**. Nodes are represented by green spheres, and connective edges by transparent white lines, with the exception of primary cluster nodes that are shown as orange spheres.

Figure 8 is a graphical representation by Cytoscape (Cline et al., 2007) of the full MGCN using all 739 available arrays. The main component of the network consists of 3394 (79.7%) of the total nodes, and 41,238 (60.3%) of the total edges. The smallest component consists of 9 nodes with 9 edges. The network also consists of 14 disconnected components, wherein each component is a pair of directly or indirectly connected nodes.

The full MGCN has a clustering coefficient of 0.435, with nodes having an average of 28.9 neighbors each. Across the total 4257 nodes, there exists 11,676,414 shortest paths, with the characteristic path length averaging 5.5 edges. As shown in Supplementary Figure 1, the relationship between node number and degree fits a power-law distribution with a tail, which is indicative of a scale-free network. By comparison, the main component of the network has a clustering coefficient of 0.396, with nodes having an average of 22.2 neighbors each. With a stringent PCC cutoff of 0.93, and the associated loss of information, only 4 of the 16 total MtARF genes were left in the final network (MtARFs 4, 10, 17, 20). Of these four genes, MtARF4 (Medtr2g005240) had the most neighbors of 14. MtARF10 (Medtr3g064050) had two neighbors, while MtARFs 17 and 21 (Medtr5g076270, Medtr8g079492) had a single neighbor each. All four MtARFs present in this “stringent” network were found within the main component of the full MGCN.

**Figure 9:**
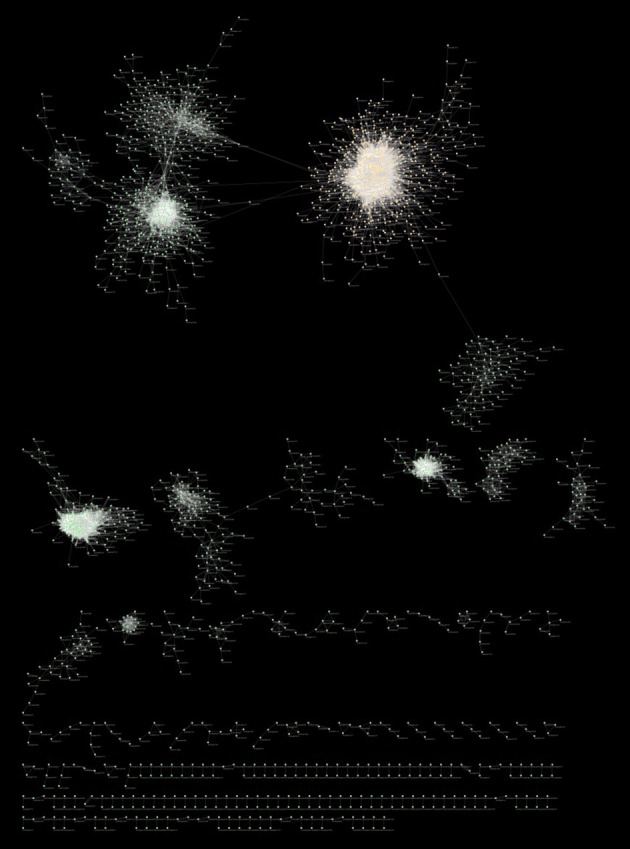
**Cytoscape visualization of the root MGCN**. Nodes are represented by green spheres, and connective edges by transparent white lines with the exception of the nodes of the primary cluster that are shown by orange spheres.

The root-only MGCN has a clustering coefficient of 0.423, with nodes having an average of 28.3 neighbors each. In contrast to the full MGCN with 14 disconnected components, the root-only MGCN consists of 161 disconnected components. A graphical representation of root-only network was also obtained using Cytoscape (Figure 9). The relationship between node number and degree again fits a power-law distribution with a tail, indicating that a scale-free network underlies the root-only dataset as well. The main component of the root-only MGCN, representing 60.2% of the whole network, contains 24,018 edges connecting 1604 nodes. The main component also shares a similar clustering coefficient and neighbor count to the rest of the network (0.412 and 26.3, respectively). Surprisingly, 6 MtARF genes were found in the root-only MGCN despite this network only having 57% of the gene population of the full MGCN. Of the 6 genes, 2 were also present in the full MGCN (MtARF4, MtARF20). All 6 MtARF genes are present in the main component, as was the case with the 4 MtARFs of the full MGCN. Interestingly, unlike the full MGCN, the degree of connectivity amongst the 6 MtARFs was drastically increased. MtARF7, which is absent in the full MGCN, has a total of 56 direct neighbors. Similarly MtARF1 that was also absent in the full MGCN has a total of 21 neighbors. Of the conserved MtARFs across both networks, MtARF4 has only a single neighbor in the root MGCN (compared to 14 in the full network) and MtARF20 has 14 neighbors in the root-only network (compared to a single neighbor in the full network). Cytoscape session files for unweighted networks are made available as Supplementary Presentations 10, 2.

Genes next to MtARF genes in MGCNs were examined for enrichment; the gene descriptions were retrieved from the JCVI *M. truncatula* annotation database (Town, 2006). Leucine-rich receptor-like kinase and topless-like proteins were the most recurrent neighbor types (4), followed by transcription factor jumonji (JmjC) domain proteins (3). Significant co-expression of MtARF10 and MtARF18 was found in both the full and root MGCNs. No other member of the MtARF family showed significant co-expression in either network. Notably, MtARF4 had a unique decrease in connectivity in the root network relative to the full network; we found a singular serine/threonine kinase family protein among the 14 neighbors of MtARF4 in the full MGCN. None of the 14 neighbors of MtARF20 in the root MGCN were connected to MtARF20 in the full MGCN.

### Network clustering

While elucidating direct MtARF neighbors in MGCNs and their potential functions may be indicative of the possible roles of an MtARF gene, it is important to leverage the modular nature of biological networks to decipher the functions of MtARF genes in larger metabolic contexts. To do so, one can employ a variety of clustering tools to isolate modules from the entire network, and investigate the functional enrichment of modules harboring the MtARF genes. We chose the MCL algorithm to partition the MGCN networks because of its past success in application to different biological networks (Brohée and van Helden, 2006).

Markov clustering using the MCL algorithm was applied across both networks over a range of inflation parameter values from 1 to 5. The resulting clusters were subjected to single enrichment analysis using the AgriGO interface. The cluster configurations obtained at different inflation parameter values were compared in order to identify biologically-relevant configurations (Du et al., 2010). Briefly, the clusters obtained for each tested inflation value were analyzed for term enrichment. The inflation parameter yielding the highest number of clusters with enriched terms, as well as the most terms per cluster was deemed the optimal value. This led to an optimal inflation parameter of value 1.2 for both networks, as this value resulted in highest number of clusters with term enrichment, as well as highest number of terms per enriched cluster. In the full MGCN, MtARF 4 and MtARF 20 were found in the largest (or primary) cluster, whereas MtARF 10 and MtARF 17 were assigned to a small cluster for which an enrichment analysis couldn't be performed due to small sample size. For the root MGCN, all 6 MtARFs were found in the primary cluster. In addition to all four MtARFs present in the full MGCN, the root MGCN also includes MtARF 1 and MtARF 7. Enrichment analysis results for primary clusters from the full and root MGCNs were similar, with a high enrichment of intracellular and vesicle-mediated transport proteins. Ubiquitin-dependent protein catabolic processes and their parent terms were significantly enriched (FDR < 0.05) in both networks; the full MGCN differed only in the additional enrichment of the regulation of Ras protein signal transduction. The cellular component enriched terms were also similar for the full and root-only MGCNs' primary clusters, with membrane-bounded vesicles and the endomembrane system among the most prominently enriched. Molecular function enriched terms for the full MGCN's primary cluster were also similar to those for root-only MGCN, with protein and zinc-ion binding topping the enriched terms. Taken together, both primary clusters appear to represent a transcriptionally-responsive signaling pathway, and it is no surprise that the MtARF family is represented within both. Unfortunately, and possibly due to the small size of the unweighted networks, the enrichment analysis had to be restricted to rather more generic terms.

### Weighted gene co-expression network and analysis

The full MtGEA was used to construct a weighted gene co-expression network using the WGCNA method (Langfelder and Horvath, 2008). Individual probeset IDs from the arrays in the gene atlas were the network nodes, and the edges signified the connection strength between nodes. This was done to address the possibility that converting probes to gene models prior to network construction may lead to a loss of representation due to the consolidation of each probe's expression profile. As described in the Materials and Methods Section, normalized expression levels were Pearson correlated, and subsequently risen to the power 8. This value was determined as the minimum in order to generate a maximum scale-free topological fit (see Section Materials and Methods). These raised correlation values, or adjacencies, were then used to form a topological overlap matrix (TOM) to which hierarchical clustering and subsequent branch cutting were employed to obtain independent functional modules.

Altogether, 23,209 probeset IDs were grouped into modules, based on the first principle component of each seeded network module, referred to as “eigengene” by WGCNA. The correlations of gene expression pattern for each probeset ID to these eigengenes were then examined to determine the module membership of a probeset ID (Langfelder and Horvath, 2008). Twelve MtARF genes, represented by 23 different probeset IDs, were found distributed in three modules. Twenty-two of these 23 MtARF-related probeset IDs were distributed across two modules, cumulatively accounting for 11 MtARF genes. These two modules were significantly enriched for nitrogen compound metabolic processes, and post-embryonic development. In fact, all but 58 of the 789 total probes enriched for nitrogen compound metabolic processes were found in these two modules. While one of the two clusters (Supplementary Table 1) showed very general locational enrichment (“Cell,” “Cell Part”), the other (Supplementary Table 2) showed significant enrichment in the Golgi apparatus, as well as the cytoskeleton. Functional enrichment for the two clusters included DNA and nucleotide binding and phosphotransferase activity. The smallest of the three MtARF containing clusters (Supplementary Table 3) demonstrated significant enrichment for phenylpropanoid biosynthesis, electron transport, oxidoreductase activity, and heme binding. Locational enrichment typified the module as being closely associated with membrane-bounded vesicles.

### Mutual rank network and analysis

A third network-based strategy to understanding the MtARF family's regulatory nature was performed using mutual rank (MR) analysis. Ranked networks focus on the order of PCC values for each gene, and can identify genes sharing similarly ranked correlations that might otherwise be disposed of by PCC-based thresholds in typical co-expression networks. This is especially true for genes that have low maximum PCC values, which are completely excluded from such networks, which was the case for several MtARF members in the full and root MGCN. A weighted-PCC calculation was performed for every gene pair based on the sample redundancy inherent to the MtGEA (see Section Materials and Methods). Additionally, an MR cutoff of 100 was imposed in order to minimize network density without significant information loss that is often the case with the PCC-based unweighted networks.

Unlike the other network models used in this study, all 16 of the 23 MtARF genes represented in the MtGEA were present in the *M. truncatula* MR correlation network (MtMRN). Most of the direct neighbors of MtARF in the MtMRN had MR values closer to the cutoff value, with 64 (69.6%) of the MR values being higher than 70, at an average MR value of 73.8. The lowest MR value, 17, exists between MtARF6 (Medtr2g018690), and Medtr3g081140, which is described as a cysteine-rich transmembrane module stress tolerance protein according to Uniprot (Bateman et al., 2015). Medtr3g081140 also shares the second and third lowest MR value in the network with MtARF10 and MtARF5 with MR of 18 and 21 respectively.

Of the MtARF family's 92 direct neighbors in the MtMRN representing 41 different genes, 10 genes, located on chromosomes 4 and 5, encode late nodulin proteins (Pfam 07127). These 10 genes are direct neighbors of MtARFs 5, 6, 7, 10, 16, 18, 21. MtARF18 has the highest connectivity in this network, with 15 direct neighbors, followed by MtARF17 with 11 neighbors. A germin-like protein coding gene, Medtr2g086630, is the most prolific of the direct neighbors, connecting to 13 of the 16 MtARFs with a MR below 100. The second most prolific, connecting to 9 MtARFs, is the same cysteine-rich TM module stress tolerance gene that also shares the three lowest MR values with MtARFs in the MtMRN.

As with the full and root MGCN unweighted networks, MCL algorithm clustering was applied with inflation values between 1 and 5. The resulting clusters containing MtARF members were then tested for GO term enrichment using the AgriGO online analysis tool with a custom background containing only members present in the MtMRN (Du et al., 2010). The largest cluster harbored a majority (>62.5%) of MtARFs present in the MtMRN, for any inflation values between 2.5 and 4. An inflation parameter of 2.8 was chosen as this value resulted in the highest number of enriched terms in the primary cluster. Only the primary cluster was considered relevant for all tested inflation values, as all other clusters containing MtARFs did not shave any significant enrichment at any inflation parameter.

The largest cluster of the MtMRN, generated using an inflation parameter of 2.8 and an MR cutoff of 100, contained 12 of the 16 MtARFs present in the network. Three of the MtARFs not included (MtARFs 10, 18, and 6) were found in the third largest cluster, and a single MtARF (19) was found in the fifth largest cluster. Single enrichment analysis of this primary cluster displayed significant overrepresentation of many biological processes, including nucleotide metabolic process (GO: 0009117), ubiquitin-dependent protein catabolic process (GO: 0006511), tRNA processing (GO: 0008033), cellular nitrogen compound biosynthetic process (GO: 0044271), DNA repair (GO: 0006281), regulation of Ras protein signal transduction (GO: 0046578), and intracellular protein transport (GO: 0006886). Cellular component enrichment was primarily confined to intracellular non-membrane-bounded organelle (GO: 0043232), with more specific terms included the ribosome (GO: 0005840). Certain molecular functions were also significantly enriched, with protein binding (GO: 0005515), hydrolase activity (GO: 0016817, GO: 001618), ATP-dependent helicase activity (GO: 0008026), zinc ion binding (GO: 0008270), and ubiquitin thiolesterase activity (GO: 0004211). A full breakdown of all enriched terms is given in Supplementary Tables 1–7.

Analysis of the remaining clusters was less informative. The third largest cluster, containing three MtARFs, was only enriched in a single term for copper ion binding (GO: 0005507). The fifth largest cluster, containing MtARF18, was not significantly enriched with any GO term. This was similarly true for secondary clusters at varying inflation parameters.

## Summary and discussion

An important goal in plant biochemistry and molecular biology is to identify and functionally characterize the gene families that play a central role in plant physiology and metabolism. With high-throughput DNA and RNA sequencing at the helm, functional analyses must leverage these data to gain a systems-level understanding of the genes or gene families and their interacting partners, with the ultimate goal of translating the molecular database to a knowledgebase of organismal processes. Here we utilized the publically available genomic and transcriptomic data of a model plant *M. truncatula* to augment current knowledge of an important gene family. The ARF family of *M. truncatula* has been the focal point of prior investigations as well to gain a thorough understanding of its role and that of its interacting partners (Shen et al., 2015). Our analysis advances this further through a comprehensive network analysis utilizing the wealth of Next-Gen sequencing data available for *M. truncatula*. Gene networks, usually derived from co-expression patterns observed across a multitude of experimentally diverse conditions, enable visualization of interactions, and importantly, the functional characterization of genes as such networks tend to organize as functional clusters or modules reflecting the underlying pathways that govern the biological processes. Information-rich networks have been constructed for plant models, including *A. thaliana, V. vinifera*, and rice (Mao et al., 2009; Obayashi et al., 2011; Liang et al., 2014), enhancing our understanding of potential functions beyond single or handful of genes.

### MtARF genes and their expression profiles

**Figure 10:**
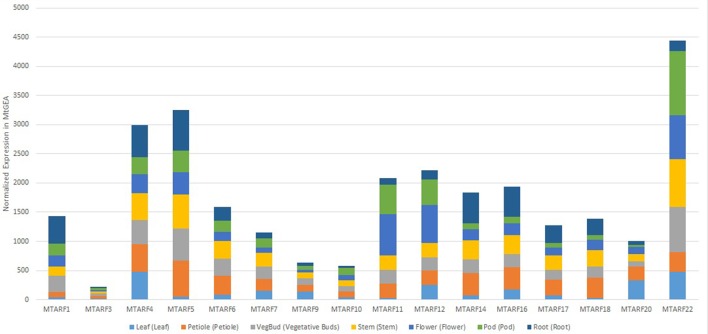
**Normalized expression of 16 MtARF genes that are represented by at least one probe in the MtGEA, shown by experimental tissue types**. Expressions are shown in terms of normalized counts in the atlas.

Over 68% of the predicted MtARF genes had a carboxy-terminal AUX-IAA domain, in addition to the characteristic B3-DNA binding and auxin-responsive domains, implying the MtARF family's role in auxin-responsive transcriptional activation. This is similar to the proportion of ARF genes with AUX-IAA domains in other plants, such as *A. thaliana* (83%) and *B. rapa* (77%; Mun et al., 2012). Our multi-pronged search approach coupled with transcriptional validation yielded 8 novel MtARF genes. Expression profiles of the 16 MtARFs represented within the MtGEA showed no preference for a singular tissue type for wild-type, untreated tissue extracts (Figure 10), though overall expression of the MtARFs varied significantly. Chromosomal position does appear to share a relationship with expression, with the four most expressed MtARF genes present in the atlas (MtARFs 4, 5, 21, and 22) being located near the telomeric regions of Chr 2 and Chr 8.

### Hierarchical relationships among MtARF gene family members

**Figure 11:**
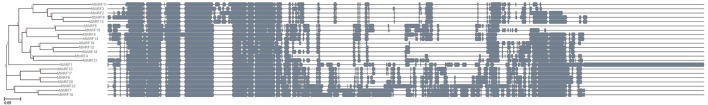
**Dendrogram constructed following multiple sequence alignment for all 22 MtARFs using Clustal Omega and ETE 3 visualization framework**.

**Figure 12:**
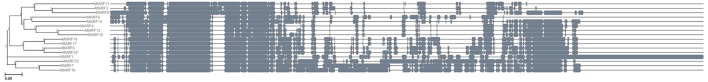
**Dendrogram constructed following multiple sequence alignment for the 16 MtARFs represented by at least one probe in the MtGEA using Clustal Omega and ETE 3 visualization framework**.

To better understand the relationships within the MtARF gene family, a heatmap based on expression data for the 16 MtARF genes represented in the MtGEA was generated using Euclidean clustering (Figure 5). Two major clades were apparent based solely on the expression profiles of these gene models across the 700+ experimental conditions of the atlas. MtARFs 2, 3, 5, 9, 11, 13, and 15 are the only family members found lacking the carboxy-terminal AUX-IAA domain. Of these 6 genes, 4 had representative probes within the MtGEA, and all four were found within a single major clade with comparatively lower expression across the entire gene atlas. Furthermore, none of the 6 genes lacking the AUX-IAA domain were enriched for glutamine in their middle regions (Table 1), which may indicate a link between lower universal expressions for MtARFs that function as transcriptional repressor. Multiple sequence alignment using Clustal Omega (Sievers et al., 2011) and visualized with the ETE 3 Framework (Huerta-Cepas et al., 2016) echoed many of the relationships established in the expression-based clustering. Most striking is the relationship between MtARF12 and MtARF18, whose expressional similarity makes a prominent band in Figure 5, and is mirrored in their sequence similarity in Figure 11. When clustering only the MtARF protein sequences for which there is representation in the MtGEA, the correlation between expression and sequence similarity becomes even more apparent (Figure 12). A tanglegram that allows visualization of concordance between the sequence and expression based dendrograms (Figures 5, 12) was obtained using Dendroscope 3.5.7 (Supplementary Figure 2).

### Functional characterization of MtARFs through multiple network approaches

A plethora of methods have been implemented to generate and cluster biological networks. The methods have been evolving faced with the challenge to decipher the exponentially growing data. Gene co-expression networks have become an increasingly popular application for deconstructing genetic wiring that underlies complex phenotypes by utilizing the data from high-throughput experiments. In general, the PCC is used under the assumption that genes with highly correlated expression patterns share functional relationships (Liu, 2015). Therefore, a primary goal of such analysis is to identify functional clusters (or modules) that represent conserved functions, through which the members of the cluster can be identified and further characterized.

The comprehensive MtGEA expression library provided an opportunity to assess three network approaches—unweighted PCC-based methodology, WGCNA R library, and a sample-weighted PCC-based mutual rank method. In addition to the full MtGEA atlas, a subset focusing only on root tissues was used for the generation of an unweighted co-expression network in order to uncover links between the members of the MtARF family and the initiation of nodulation (Breakspear et al., 2014; Shen et al., 2015). The MCL algorithm was used owing to its prior reported successes in clustering biologically-significant networks (van Dongen, 2000; Brohée and van Helden, 2006).

Early network approaches relied heavily on PCC of gene pairs, and connections were established between gene pairs meeting a pre-established PCC threshold in order to optimize the network density for a biologically significant gene clustering (Mao et al., 2009). To circumvent the loss of information associated with such hard thresholds, weighted network approaches have been developed that employ soft thresholding whereupon the correlation coefficient is raised to the lowest power leading to a network with scale-free topology (Langfelder and Horvath, 2008). More recently, sample-weighted PCC calculations (Liang et al., 2014) and the numerical ranking of correlation values have been proposed in an effort to better characterize genes with lower universal correlation values that are otherwise overlooked in co-expression networks (Obayashi et al., 2011).

While the effect of “hard-thresholds” have been discussed in prior studies (Langfelder and Horvath, 2008), the high PCC stringency of the unweighted method that results in an “optimal” MGCN was not observed in any other similarly constructed plant networks, where the cutoffs were reported to be typically between 0.7 and 0.8 (Langfelder and Horvath, 2008; Mao et al., 2009; Liang et al., 2014). To ensure that such a high cutoff is indeed indicative of the plant physiology, and not of a biased expression atlas, a network for a subset of the expression atlas representing root was also constructed (Figure 9). Using the same metrics, an equally stringent 0.93 PCC cutoff was found optimizing the root network as well. As expected, the loss of information, primarily represented by the diminished number of genes present in the network, was apparent when such a hard-threshold was used. For the full MGCN, only 4653 of the 16,991 genes present in the atlas were represented in the final network. Representation was even lower in the root MGCN, with only 2663 genes present.

The stringent cutoffs enforced by network analysis methods on both the full and root MGCN limited the representation of the MtARF family within each. Enrichment analysis of MtARF-containing clusters revealed striking similarities in enrichment across the networks considered, and demonstrate, at least for a few members escaping the hard-thresholding effect, the transcriptional regulatory roles related to the endomembrane system, with an emphasis on signal transduction through vesicle-mediated transport. A more precise characterization of the family was obtained through generation of a WGCNA-generated network, where 12 of the 16 MtARFs present in the MtGEA were represented. With probes associated with 11 of the 12 MtARFs falling within two functionally similar clusters, additional enrichment terms associated with nitrogen compound metabolic processes and post-embryonic development were discovered in relation to the gene family. The vesicular transport and endomembrane localization terms of the MGCN networks were reinforced by the WGCNA-based network, lending credence to the MtARF family's known role as a highly ubiquitous transcriptional regulator, and reflecting known relationships between certain MtARFs and auxin transporters such as the PIN family (Sauer et al., 2006).

The MtMRN was the only network to include all 16 of the MtARF genes of the MtGEA, with 75% of the members falling into a single cluster. The larger size of this network, with 8166 genes contributing to a mutual rank of 100 or lower with at least one partner gene, allowed for more inclusive clustering that resulted in many functionally significant clusters as revealed by the enrichment analysis. The MtMRN's primary cluster, harboring 12 of the 16 MtARF family members, was found to be a transcriptionally responsive collection of genes, with the majority of genes associated with the ribosome, helicase activity, DNA repair, and glycolysis. Ras protein signal transduction was also enriched, echoing its presence from the enrichment analysis of primary clusters in the full and root MGCNS, as well as the weighted WGNCA-based network.

Direct neighbor analysis of this more inclusive network, specifically the direct neighbors of MtARF genes, revealed a potentially ubiquitous family of transcriptional regulators. A total of 41 direct neighbors were found. Late nodulin proteins, containing the Pfam 07127 Nodulin_late domain, comprised 10 (24%) of all MtARF direct neighbors in the MtMRN. With a threshold of MR <= 100, direct neighbors within the MtMRN were expected to share not just significant co-expression pattern, but they were also expected to be similarly prioritized relative to all other genes within the network. This helped avoid potentially misleading connections between the genes in a network. The enrichment analysis of the MtMRN's primary cluster, a collection of over 4874 genes, suggests its central role in proper nodule development, with significant transcriptional regulation carried out by the members of this cluster. Furthermore, all three network strategies resulted in clusters that were enriched in transport-centric and signaling genes, revealing how auxin exhibits control throughout the entire plant system. The differences in direct MtARF neighbors between the full and root MGCNs may provide insights into auxin's seemingly opposite transcriptional activities in the root compared to other plant tissues through related transcription factors. All three network approaches in this study point toward a cluster of highly-interrelated genes, encoding a protein assembly and secretory pathway that is regulated, in part, by a family of auxin-responsive transcriptional factors. Mutually-ranked network of co-expressed genes provided much more detail and specificity in the significantly enriched functional terms. MtMRN appeared more efficient in deciphering a highly-correlated expressome compared to other network models.

It appears from previous studies focusing on ARF inhibition, and in particular the auxin homeostasis and nodule development, that several relatively well-characterized members of the MtARF family play key role in regulation of nodulation. The inhibition of auxin transporters has been shown to regulate the expression of nodulin genes and thereby induce nodule-like structures; it is therefore not surprising how commonplace nodulin genes appear to be among the direct neighbors of MtARF genes in the MtMRN (Bustos-Sanmamed et al., 2013). However, the mechanism of regulation, beyond the presence or absence of auxin, remains poorly understood. The auxin associated regulatory network is vast, possibly involving thousands of intricately connected genes (Mathesius et al., 1998; Hagen and Guilfoyle, 2002; Suzaki et al., 2013). Over 1500 genes were reported to underlie the transcriptional initiation of nodules (Breakspear et al., 2014), whilst the primary clusters containing MtARF members in this study approached 5000 genes. Given a multitude of roles, many still unknown, that are likely played by this regulatory framework, a systems-level analysis, such as a network approach as demonstrated in this study, will go a long way in deciphering the full spectrum of genes within this network or associated pathways. Understanding the mechanisms that govern the auxin transport to the nodule proliferation is being made possible by the advances in sequencing as well as network biology and further efforts on integrating diverse data are needed to decipher molecular factors regulating symbiosis.

It is expected that the genomic and transcriptomic database will be rapidly and continually enriched enabling construction of even more robust networks for the functional characterization of genes and gene families. Beyond the characterization of a single gene family, the networks established in this study may provide valuable insights into the uncharacterized genes. At the time of this study, 841 of the 8533 genes present in the primary cluster of the MtMRN were uncharacterized according to the Phytozome 11 Phytomine database for *M. truncatula* (Goodstein et al., 2012). Given what is currently known about their highly-correlated network neighbors, it may be any number of these genes that holds the key to reconstructing the pathways or subnetworks that are governed by the actions of auxin.

## Author contributions

DB and RA conceived and designed the experiments. DB performed the experiments. DB and RA analyzed the data and wrote the manuscript. DB and RA read and approved the final manuscript.

## Funding

This work was supported by a faculty start-up fund from the University of North Texas to RA and a Beth Baird graduate student scholarship to DB.

### Conflict of interest statement

The authors declare that the research was conducted in the absence of any commercial or financial relationships that could be construed as a potential conflict of interest.

## Supplementary material

Supplementary Figure 1 **Distribution of node degree for the full MGCN shown on log scale**. The power-law fit (red line) to the distribution is indicative of a scale-free network (see text).

Supplementary Figure 2 **Tanglegram with the expression dendrogram (left) and the sequence dendrogram for the MtARF family**.

Supplementary Tables 1–3 **GO enrichment of the three clusters containing MtARFs in the WGCNA network**.

Supplementary Table 4 **GO enrichment of the primary (largest) cluster of the full MGCN using a MCL inflation parameter of 1.2**.

Supplementary Table 5 **GO enrichment of the primary (largest) cluster of the root MGCN using a MCL inflation parameter of 1.2**.

Supplementary Table 6 **GO enrichment of the primary (largest) cluster of the mutual rank network using a MCL inflation parameter of 2.8**.

Supplementary Table 7 **Transcriptomic analysis of the 44 potential MtARF genes using 36 publically available RNA-Seq datasets from the NCBI SRA with included accession numbers**. Values for each replicate are in FPKM.

Supplementary Cytoscape session File 1 **Full_Network_Cytoscape_MGCN_93.cys (Presentation 1)**.

Supplementary Cytoscape session File 2 **Root_Network_Cytoscape_MGCN_93_Roots.cys (Presentation 2)**.
