# Supplementary figures and images for: Identification and Network-Enabled Characterization of Auxin Response Factor Genes in Medicago truncatula
Source: Front Plant Sci. 2016 Dec 9;7:1857. doi: 10.3389/fpls.2016.01857 (PMC5145899; doi:10.3389/fpls.2016.01857)

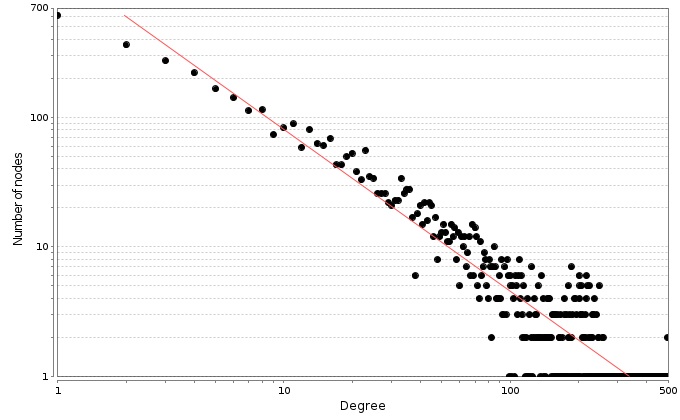

Supplement: Supplementary Figure 1 — Distribution of node degree for the full MGCN shown on log scale. The power-law fit (red line) to the distribution is indicative of a scale-free network (see text). [file Image1.JPEG]

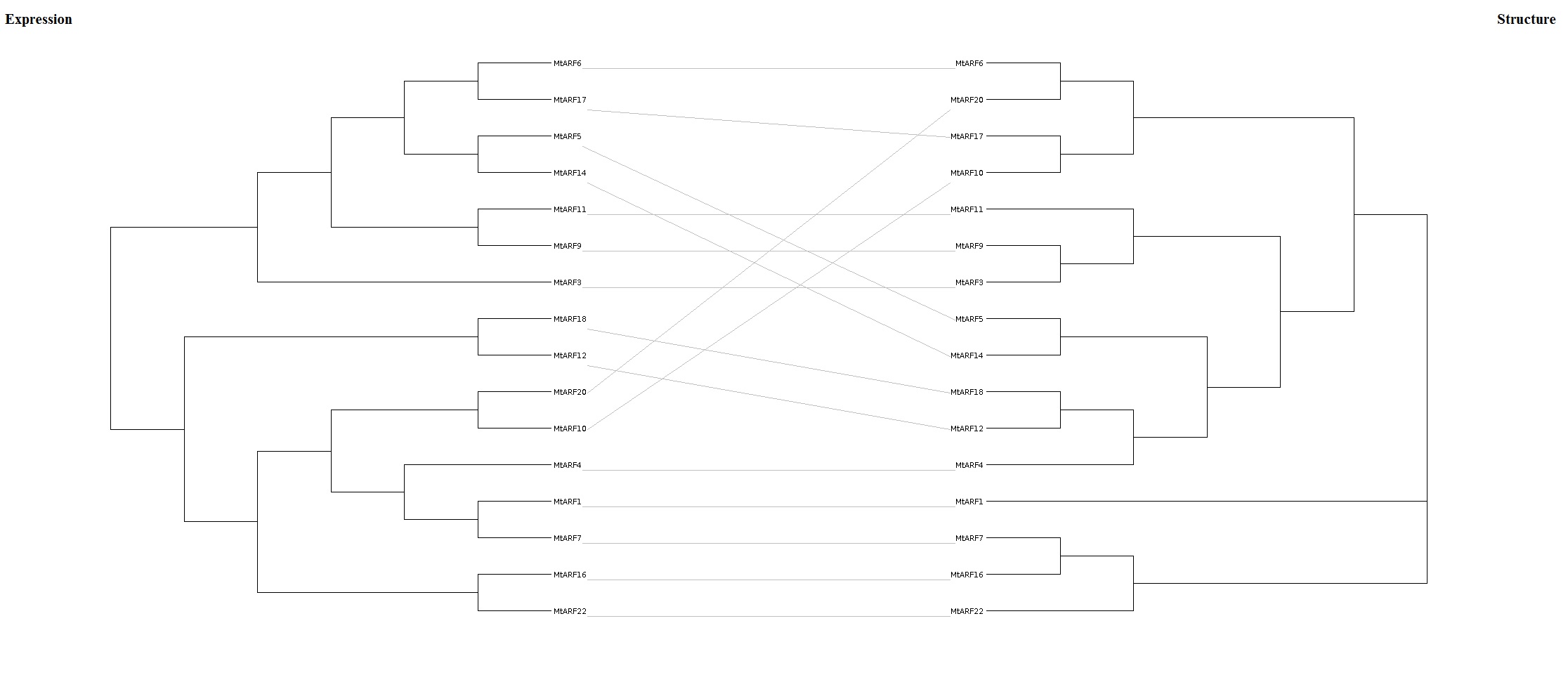

Supplement: Supplementary Figure 2 — Tanglegram with the expression dendrogram (left) and the sequence dendrogram for the MtARF family. [file Image2.JPEG]
